# Which Recurrent Selection Scheme To Improve Mixtures of Crop Species? Theoretical Expectations

**DOI:** 10.1534/g3.119.400809

**Published:** 2019-10-31

**Authors:** Jean-Paul Sampoux, Héloïse Giraud, Isabelle Litrico

**Affiliations:** INRA, Centre Nouvelle-Aquitaine-Poitiers, UR4 (UR P3F - Unité de Recherche Pluridisciplinaire Prairies et Plantes Fourragères), CS80006, 86600 Lusignan, France

**Keywords:** Progeny family test, Response to selection, Reciprocal mixture ability, General mixture ability, Selection index

## Abstract

In a context of increasing environmental challenges, there is an emerging demand for plant cultivars that are adapted to cultivation in species mixture. It is thus pressing to look for the optimization of selection schemes to grow species mixtures, and especially recurrent selection schemes which are at the core of the improvement of many plant species. We considered the case of two populations from different species to be improved by recurrent selection for their performances in mixture. We set up an analytical model of performances in mixture. We expressed the expected responses of the performances in mixture to one cycle of selection in the case of a Reciprocal Mixture Ability selection scheme and of two parallel selection schemes aiming to improve General Mixture Abilities or performances in pure stands. We numerically compared these selection schemes when half-sib or topcross progeny families of selection candidates are tested in mixture. Selection in pure stands appeared efficient within a limited range of genetic correlations between pure stand performance and mixture model effects. The Reciprocal Mixture Ability selection scheme was expected to be less efficient than parallel selections for General Mixture Ability in some situations. The last option enables to control the ratio of expected responses of species contributions to the mixture performance without bias when using selection indices. When more than two species are be improved for their performances in mixture, the advantage of parallel selections for General Mixture Ability is even more marked, providing that compensation trends between species are not too prevalent.

Many studies have contributed to demonstrate the asset of species diversity on the stability of plant production systems (Allard 1961; Tilman *et al.* 1996; Hector *et al.* 1999; Finckh *et al.* 2000; Grime 2006; Nyfeler *et al.* 2009). This is particularly the case under conditions of climate variability (Tilahun 1995; Lesica and Allendorf 1999; Tilman *et al.* 2001) and environmental challenge (Frey and Maldonado 1967; Hajjar *et al.* 2008; Vlachostergios *et al.* 2011; Bargaz *et al.* 2017; Raseduzzaman and Jensen 2017; Helgadóttir *et al.* 2018). Despite the recognized benefits of diversity, present plant production systems are mostly monospecific. Faced with the challenges of environmental sustainability and adaptation of plant production systems to climate change, it is worthwhile to develop an approach of plant production stability based on diversity (Tilman *et al.* 2001). However, the use of cultivated species in plurispecific mixtures would require the delivery of plant cultivars adapted to this practice, and consequently the adaptation of plant selection schemes to this objective.

Experimental results indicated that the performances of plant cultivars tested in pure stands were often moderately correlated with their performances in species mixtures. This was noticed when the pure stand performances were recorded in space-plant conditions (Atwood and Garber 1942; Caradus *et al.* 1989) as well as when they were recorded in dense stands similar to those used in standard farming conditions (Dijkstra and De Vos 1972; de Oliveira Zimmermann 1996; Holland and Brummer 1999; Santalla *et al.* 2001; Gebeyehu *et al.* 2006). It is thus clear that breeding plant cultivars for their performances in pure stands may not always be an efficient way to create cultivars adapted for usage in species mixtures and that selection schemes especially designed to improve performances in species mixtures should be looked for (Fyfe and Rogers 1965; Dijkstra and De Vos 1972; Hamblin *et al.* 1976; Davis and Woolley 1993; de Oliveira Zimmermann 1996; O’Leary and Smith 2004; Gebeyehu *et al.* 2006; Annicchiarico and Proietti 2010). To our knowledge, no breeding experiments have so far practically compared the efficiency of selection in pure stands and of selection methods especially designed for inter-specific objectives in order to improve performances in mixtures. Such studies would indeed require very long term projects and need weighty protocols. Past experimental assessments of recurrent selection methods applied to pure stand performances of plant species have used at least four to eight selection cycles to reveal consistent trends of improvement (*e.g.*, Russell *et al.* 1973; Compton and Bahadur 1977; Moll and Hanson 1984; Eyherabide and Hallauer 1991; Ramalho *et al.* 2005).

The theory of selection in plant breeding provides the conceptual framework for an *a priori* comparison of selection methods. This framework has long been used to compare plant selection methods for their efficiency to improve performances in pure stands (*e.g.*, Allard 1960; Hanson and Robinson 1963; Sprague 1966; Mayo 1980; Hallauer and Miranda 1981; Gallais 1989). It has indeed efficiently guided practical selection efforts in the development of modern plant breeding, even if its implementation has often required a radical simplification of the objectives that plant breeders have to target. Theoretical developments have especially been extensive for the optimization of recurrent selection methods targeted to improve performances in pure stands (*e.g.*, Comstock *et al.* 1949; Griffing 1963; Cress 1966; Hallauer and Eberhart 1970; Jones *et al.* 1971; Compton and Comstock 1976; England 1977; St. Martin 1986). With the objective of improving performances in species mixtures, Wright (1985) proposed a recurrent reciprocal scheme for two species in which each progeny family from one species was tested in mixture with a progeny family from the other species. The best performing progeny family pairs from this reciprocal design were to be selected to generate the next cycle population in each species and to derive improved mixtures for farming usage. Wright (1985) expressed the responses of the performance of the mixture and of the contribution of each species to the performance of the mixture expected from one cycle of selection before recombination of selected candidates according to several variations of the breeding objectives and generalized the expression of these expected responses in the case where more than two species were to be improved for their performances in mixture.

Furthermore, it has been proposed by different authors (*e.g.*, Williams 1962; Gallais 1970; Jacquard *et al.* 1978; Wright 1985) that the performance of a mixture of genetic units (cultivars or progeny families) from different species be modeled as the sum of their General Mixture Abilities (GMAs) and their Specific Mixture Ability (SMA). The GMA of a given genetic unit is its average performance in mixture with any of the genetic units from the other species, while the SMA of a specific mixture is defined as the difference between the observed performance of this particular mixture and its performance predicted from the sum of the GMAs of its components. GMA and SMA are also known in plant ecology as general and specific ecological combining abilities (Harper 1967). Using GMA and SMA as breeding criteria could indeed be seen as attractive by plant breeders. More specifically, it could be tempting to set up selection schemes designed to improve GMAs in populations (Hill 1990). Such selection schemes could be practically easier to implement than the reciprocal scheme suggested by Wright (1985). In addition, improving the sole performance of the species mixture is generally not the only breeding goal to target. The contributions of the different species making up a mixture should usually stay within some proportions to benefit from the agronomic and agro-ecological assets of species mixture (Annicchiarico and Piano 1994; Corre-Hellou *et al.* 2006) and possibly to maintain sufficient economic value or technical quality of the mixture products (Thomas 1992; Lüscher *et al.* 2014). However, it was clearly documented that dominant components in a mixture often grow at the expense of less agressive ones (Connell 1983; Zannone *et al.* 1986; Corre-Hellou *et al.* 2006; Annicchiarico and Proietti 2010; Lithourgidis *et al.* 2011; Boukar *et al.* 2015; Annicchiarico *et al.* 2017; Brophy *et al.* 2017). Compensating trends can thus set in, in which changes in the genetic composition of the mixture components can induce substantial changes in their proportions (Williams 1962; Norrington-Davies 1967; Gallais 1970; Breese and Hill 1973; Gleeson and McGilchrist 1978). Accordingly, it should be necessary that selection methods designed to improve the performances of species mixtures also include some means to control the responses of the contributions of the different species making up the mixture.

In this paper, we considered the case of two populations from different species that should be improved by recurrent selection for their performances in mixture. We considered that the overall performance of the species mixture was the main trait to improve. We expressed the expected response to selection of the performance of the species mixture and of species contributions to the performance of their mixture after one cycle of selection using two different selection schemes: (i) the reciprocal scheme proposed by Wright (1985) and (ii) an alternative scheme based on the parallel improvement of the two populations for their General Mixture Abilities. We also expressed the expected correlative responses of the performance of the mixture and of species contributions to the performance of the mixture from one cycle of parallel recurrent selections in pure stands in the two populations. We numerically compared responses to selection expected from these three selection methods by varying the variance-covariances of model effects included in the usual analytical model of the performances of plant mixtures (Williams 1962; Griffing 1967; Gallais 1970; Wright 1985). We furthermore investigated a way to control the responses of species contributions to the performance of the mixture using selection indices and assessed the efficiency of using such indices within the same range of variation of variance-covariances of mixture model effects. We also briefly discussed the case where more than two species should be improved for the performance of their mixture and drew some lessons regarding this case.

## Theory and Methods

We considered several recurrent selection methods applied to two populations from different species in order to improve the performance of their mixture for a single phenotypic trait, which value can be obtained as the sum of its measurement on the two species. This may be for example the case of pluri-specific sown forage meadows whose usage value primarily depends on the total biomass yield of the meadow (*i.e.*, the sum of the biomasses of all species components of the meadow harvested at the same time). In the case of intercrops for which the two species are harvested separately, the single trait to improve can be the total economic value of the mixture, set as the sum of the economic values of the products harvested on each species (which may depend on several phenotypic traits for each species). The recurrent selection methods we compared are based on the test of progeny families of selection candidates in each species. We compared the different methods on the basis of responses to selection expected from one cycle of selection. For each species, we assumed that candidate genotypes selected at cycle *n* were intercrossed in panmictic equilibrium to generate the cycle *n+1* population. We assumed a disomic inheritance and that epistasis effects were negligible. Under these assumptions, the change in population mean value from cycle *n* to cycle *n+1* is only due to the effect of selection (Gallais 1989). We established general expressions of expected responses to selection that apply whatever type of progeny families is used for testing (half-sib or full-sib progeny families, topcross progeny families, inbred progeny families) and we then set up more specific expressions of these expected responses when half-sib or topcross progeny families are used. Recurrent selection including the panmictic intercrosssing of selected candidates is of course easier to implement with allogamous species and testing half-sib or topcross progeny families is almost exclusively possible with allogamous species. However, our theoretical developments do not need assumptions on the mode of sexual reproduction of species.

### Selection for reciprocal mixture ability (SRMA)

#### Selection scheme:

**Figure 1:**
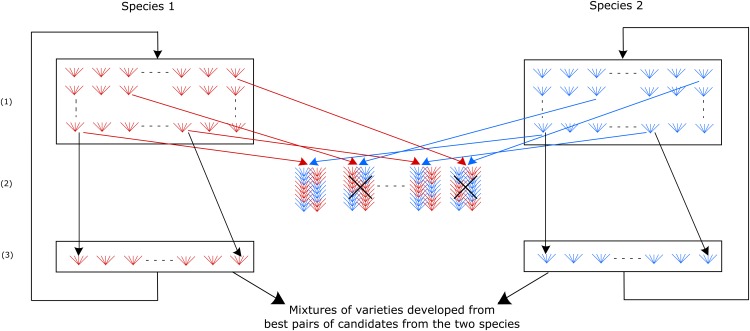
Recurrent selection for Reciprocal Mixture Ability (SRMA) in two species. (1): Populations of selection candidates at cycle *n*, (2): Experimental evaluation of mixtures of pairs of progeny families of selection candidates, (3): Recombination of the selected candidates.

This selection scheme (Figure 1) corresponds to the Reciprocal Mixture Ability selection scheme proposed by Wright (1985) for the reciprocal improvement of the mixture performance of two populations from different species. At each recurrent cycle, a joint selection is carried out in the two species based on the performance of mixtures of pairs of progeny families of selection candidates from each species. In each species, candidates from pairs selected at cycle *n* are recombined to generate the cycle n+1 population. At each selection cycle, the progeny families from outstanding pairs can also be increased and mixed to create performing mixtures for farming usage.

#### Analytical model of performances in mixture:

Consider the mixture of the progeny families from the selection candidates *r* of species 1 and *s* of species 2. The performance of the $m^{th}$ observation of this mixture is:

$$y_{1r2sm}=x_{1rm}+x_{2sm},$$

where $x_{1rm}$ and $x_{2sm}$ are the contributions to the performance of the mixture of the progeny families of candidates *r* of species 1 and *s* of species 2, respectively.

Using the same notations as Wright (1985), $x_{1rm}$ can be modeled as:

$$x_{1rm}=u_{1}+v_{1r}+a_{2s}+{\left(va\right)}_{1r2s}+e_{1rm},$$

where $u_{1}$ is the average effect of species 1 in mixture with species 2,

$v_{1r}$ is the direct effect of the progeny family of the candidate *r* of species 1 on the contribution of species 1 to the performance of the mixture with $v_{1r}\to \left(0,\sigma_{v_{1}}^{2}\right)$,

$a_{2s}$ is the associate effect of the progeny family of the candidate *s* of species 2 on the contribution of species 1 to the performance of the mixture with $a_{2s}\to \left(0,\sigma_{a_{2}}^{2}\right)$,

${\left(va\right)}_{1r2s}$ is the direct × associate interaction effect specific to the progeny families of candidates 1r and 2s on the contribution of species 1 to the performance of the mixture with ${\left(va\right)}_{1r2s}\to \left(0,\sigma_{{\left(va\right)}_{12}}^{2}\right)$

and $e_{1rm}$ is the residual effect with $e_{1rm}\to \left(0,\sigma_{e_{1}}^{2}\right)$.

Similarly, $x_{2sm}$ can be modeled as:

$$x_{2sm}=u_{2}+v_{2s}+a_{1r}+{\left(va\right)}_{2s1r}+e_{2sm}.$$

$y_{1r2sm}$ can also be modeled in terms of General Mixture Abilities (GMAs) and Specific Mixture Ability (SMA):

$$y_{1r2sm}=u_{1}+u_{2}+g_{1r}+g_{2s}+d_{1r2s}+\epsilon_{1r2sm},\text{where}$$

$g_{1r}=v_{1r}+a_{1r}$ and $g_{2s}=v_{2s}+a_{2s}$ are the GMAs of progeny families of candidates *r* of species 1 and *s* of species 2, respectively, and

$d_{1r2s}={\left(va\right)}_{1r2s}+{\left(va\right)}_{2s1r}$ is the SMA of the mixture of the progeny families of candidates *r* of species 1 and *s* of species 2

$$\text{and}\;\epsilon_{1r2sm}=e_{1rm}+e_{2sm}.$$

#### Definition of a selection criterion:

Progeny family pairs can be selected using as selection criterion a linear selection index that separately weights the contributions of the two species to the observed performance of their mixture. For a mixture combining the progeny families of candidates *r* of species 1 and *s* of species 2, such an index can be set as $I_{1r2s.}=\alpha_{1}x_{1r.}+\alpha_{2}x_{2s.}$.

The variance of this index is:

$$\begin{array}{l} \sigma_{I_{1r2s.}}^{2}=\alpha_{1}^{2}\sigma_{x_{1r.}}^{2}+\alpha_{2}^{2}\sigma_{x_{2s.}}^{2}+2\alpha_{1}\alpha_{2}Cov\left(x_{1r.},x_{2s.}\right)\\ =\alpha_{1}^{2}(\sigma_{v_{1}}^{2}+\sigma_{a_{2}}^{2}+\sigma_{{\left(va\right)}_{12}}^{2})+\alpha_{2}^{2}(\sigma_{v_{2}}^{2}+\sigma_{a_{1}}^{2}+\sigma_{{\left(va\right)}_{21}}^{2})+2\alpha_{1}\alpha_{2}(Cov(v_{1},a_{1})+Cov(v_{2},a_{2})\\ +Cov\left({\left(va\right)}_{12},{\left(va\right)}_{21}\right)+\frac{1}{M}\left(\alpha_{1}^{2}\sigma_{e_{1}}^{2}+\alpha_{2}^{2}\sigma_{e_{2}}^{2}+2\alpha_{1}\alpha_{2}Cov\left(e_{1\mathrm{rm}},e_{2sm}\right)\right) \end{array}$$

where M is the number of observations of each pair of progeny families.

If $\alpha_{1}=\alpha_{2}=1$, then $I_{1r2s.}=y_{1r2s.}$. This corresponds to the case where the selection criterion of progeny family pairs is simply the observed performance of their mixture. Its variance can be written as:

$$\sigma_{y_{1r2s.}}^{2}=\sigma_{g_{1}}^{2}+\sigma_{g_{2}}^{2}+\sigma_{d_{12}}^{2}+\frac{1}{M}\sigma_{\epsilon_{12}}^{2},\;\text{with}\;\epsilon_{1r2sm}\to \left(0,\sigma_{\epsilon_{12}}^{2}\right)$$

where $\sigma_{g_{1}}^{2}$ and $\sigma_{g_{2}}^{2}$ are the variances of GMAs in species 1 and 2, respectively, and $\sigma_{d_{12}}^{2}$ is the variance of SMA between species 1 and 2.

#### General expressions of expected responses to selection whatever type of progeny families is used:

Assuming that additive × additive epistasis effects are negligible, the expected response to selection of the mixture performance from cycle *n* to cycle *n+1* depends on the covariance between the value of the selection criterion observed for the mixtures tested at cycle *n* and the average additive genetic values inherited by offsprings of selected candidates at cycle *n+1*. The expected response to selection of the mixture performance can be expressed as:

$$\Delta G^{R}=\theta_{1}\frac{i_{1}}{\sigma_{I_{1r2s.}}}Cov\left(I_{1r2s.},A_{g_{1r}}^{T_{1}}\right)+\theta_{2}\frac{i_{2}}{\sigma_{I_{1r2s.}}}Cov\left(I_{1r2s.},A_{g_{2s}}^{T_{2}}\right),$$

the expected response of the contribution of species 1 to the performance of the mixture as:

$$\Delta G_{x_{1}}^{R}=\theta_{1}\frac{i_{1}}{\sigma_{I_{1r2s.}}}Cov\left(I_{1r2s.},A_{v_{1r}}^{T_{1}}\right)+\theta_{2}\frac{i_{2}}{\sigma_{I_{1r2s.}}}Cov\left(I_{1r2s.},A_{a_{2s}}^{T_{2}}\right)$$

and that of species 2 as:

$$\Delta G_{x_{2}}^{R}=\theta_{1}\frac{i_{1}}{\sigma_{I_{1r2s.}}}Cov\left(I_{1r2s.},A_{a_{1r}}^{T_{1}}\right)+\theta_{2}\frac{i_{2}}{\sigma_{I_{1r2s.}}}Cov\left(I_{1r2s.},A_{v_{2s}}^{T_{2}}\right)$$

where $i_{1}$ (alternatively $i_{2}$) is the selection intensity (selection differential in unit of standard deviation of the selection criterion $I_{1r2s.}$) applied to species 1 (alternatively species 2),

$\theta_{1}$ (alternatively $\theta_{2}$) equals 1 or 2 according to whether selection applies to one or two sexes in species 1 (alternatively species 2), respectively,

$A_{g_{1r}}^{T_{1}},A_{v_{1r}}^{T_{1}}$ and $A_{a_{1r}}^{T_{1}}$ (alternatively $A_{g_{2s}}^{T_{2}},A_{v_{2s}}^{T_{2}}$ and $A_{a_{2s}}^{T_{2}}$) are the average additive genetic values inherited for $g_{1r},v_{1r}$ and $a_{1r}$ (alternatively $g_{2s},v_{2s}$ and $a_{2s}$), respectively, by the offsprings of candidate 1r (alternatively 2s) at cycle *n+1* when evaluated in the same conditions of progeny mixture as candidate 1r (alternatively 2s) at cycle *n*,

and *T1* (alternatively *T2*) refers to the type of progeny families used to test candidates in species 1 (alternatively species 2).

$\theta_{1}$ (or $\theta_{2})=2$ corresponds to the case displayed in Figure 1 in which candidates selected in a species are intercrossed to generate the cycle n+1 population of this species; it also corresponds to the case in which progeny families from selfing of candidates (S1 progeny families) are intercrossed with sufficient numbers to avoid drift. $\theta_{1}$ (or $\theta_{2})=1$ corresponds to the case in which half-sib progeny families of selected candidates are intercrossed to make the cycle n+1 population. By construction, $\Delta G^{R}=\Delta G_{x_{1}}^{R}+\Delta G_{x_{2}}^{R}$. Note that interaction effects ${\left(va\right)}_{1r2s}$ and ${\left(va\right)}_{2s1r}$ are not inherited at cycle *n+1* since the pairs of progenies that will be assessed at this next cycle will again be set up at random.

#### Expected responses to selection when half-sib or topcross progeny families are used:

These two types of progeny families provide a simple situation in which the covariances between the selection criterion and the additive genetic value of mixture model effects inherited at cycle n+1 can be expressed as a weighted sum of variance-covariances of the mixture model effects at cycle *n*. If the progeny families used in species 1 for testing in mixture are half-sib progeny families, $\sigma_{v_{1}}^{2}$ is the genetic variance between half-sib progeny families for the direct effect $v_{1}$. If additive × additive epistasis is negligible, $\sigma_{v_{1}}^{2}=1/4\sigma_{A_{v_{1}}}^{2}$ where $\sigma_{A_{v_{1}}}^{2}$ is the additive genetic variance in population 1 for $v_{1r}$ and $Cov\left(v_{1r},A_{v_{1r}}^{T_{1}}\right)=1/4\sigma_{A_{v_{1}}}^{2}$. Topcross progeny families are families of offsprings from the cross of selection candidates with a unique ’tester’ genotype. If the progeny families used for testing in mixture are topcross progeny families and additive × additive epistasis effects are negligible, the genetic variance between progeny families ($\sigma_{v_{1}}^{2}$) is additive and, assuming that the tester is unchanged from one selection cycle to the next one, $Cov\left(v_{1r},A_{v_{1r}}^{T_{1}}\right)=1/2\sigma_{v_{1}}^{2}$. See Hallauer and Miranda (1981) or Gallais (1989) for the expressions of parent-offspring covariances when half-sib or topcross progeny families are used for testing. The preceding also holds true for parent-offspring covariances involving other direct and associate mixture model effects. Using these two types of progeny families, the expected response of the contribution of species 1 to the performance of the mixture can then be written as:

$$\Delta G_{x_{1}}^{R}=\theta_{1}\varphi_{1}\frac{i_{1}}{\sigma_{I_{1r2s.}}}\left(\alpha_{1}\sigma_{v_{1}}^{2}+\alpha_{2}Cov\left(v_{1},a_{1}\right)\right)+\theta_{2}\varphi_{2}\frac{i_{2}}{\sigma_{I_{1r2s.}}}\left(\alpha_{1}\sigma_{a_{2}}^{2}+\alpha_{2}Cov\left(v_{2},a_{2}\right)\right)$$

and that of species 2 as:

$$\Delta G_{x_{2}}^{R}=\theta_{1}\varphi_{1}\frac{i_{1}}{\sigma_{I_{1r2s.}}}\left(\alpha_{1}Cov\left(v_{1},a_{1}\right)+\alpha_{2}\sigma_{a_{1}}^{2}\right)+\theta_{2}\varphi_{2}\frac{i_{2}}{\sigma_{I_{1r2s.}}}\left(\alpha_{1}Cov\left(v_{2},a_{2}\right)+\alpha_{2}\sigma_{v_{2}}^{2}\right)$$

where $\varphi_{1}$ (alternatively $\varphi_{2}$) which equals 1 or 1/2 according to whether the progeny families tested in species 1 (alternatively species 2) are half-sib or topcross progeny families, respectively.

The variance-covariances of direct and associate effects are however not assessable if the progeny families from each species are tested in mixture with a single progeny family from the other species, or they are not assessable with sufficient accuracy if the progeny families of each species are tested with only a small number of progeny families from the other species. In such situations, the preceding expectations of responses to selection after recombination of selected candidates are not assessable or assessable with insufficient accuracy.

#### Expected responses to selection before recombination of selected candidates:

Whatever the type of progeny families and the pairing design of these families, it remains possible to assess the expected responses to selection before recombination of selected candidates, provided that the same selection intensity is applied in both species (which is notably the case if the progeny families of each species are tested in mixture with a single progeny family from the other species):

$$\delta G_{x_{1}}^{R}=\frac{i}{\sigma_{I_{1r2s.}}}Cov\left(I_{1r2s.},G_{x_{1}}\right)=\frac{i}{\sigma_{I_{1r2s.}}}\left(\alpha_{1}\sigma_{G_{x_{1}}}^{2}+\alpha_{2}Cov\left(G_{x_{1}},G_{x_{2}}\right)\right)$$

and

$$\delta G_{x_{2}}^{R}=\frac{i}{\sigma_{I_{1r2s.}}}Cov\left(I_{1r2s.},G_{x_{2}}\right)=\frac{i}{\sigma_{I_{1r2s.}}}\left(\alpha_{1}Cov\left(G_{x_{1}},G_{x_{2}}\right)+\alpha_{2}\sigma_{G_{x_{2}}}^{2}\right)$$

where $G_{x_{1}}$ and $G_{x_{2}}$ are the genetic components of $x_{1r.}$ and $x_{2s.}$, respectively.

Note that if the selection criterion is the performance of the mixture ($y_{1r2s.}$) and if half-sib or topcross progeny families are used and $\theta_{1}\varphi_{1}=\theta_{2}\varphi_{2}=1$, $\delta G_{x_{1}}^{R}-\Delta G_{x_{1}}^{R}=\frac{i}{\sigma_{y_{1r2s.}}}\left(\sigma_{{\left(va\right)}_{12}}^{2}+Cov\left({\left(va\right)}_{12},{\left(va\right)}_{21}\right)\right)$ and $\delta G_{x_{2}}^{R}-\Delta G_{x_{2}}^{R}=\frac{i}{\sigma_{y_{1r2s.}}}\left(\sigma_{{\left(va\right)}_{21}}^{2}+Cov\left({\left(va\right)}_{12},{\left(va\right)}_{21}\right)\right)$.

#### Tuning the selection index to meet a targeted ratio of expected responses of species contributions:

The contributions of the two species to the performance of the mixture usually have to be maintained within certain proportions. The index weights have thus to be tuned in order to control the expected responses to selection of the species contributions. This can be achieved by choosing the index weights $\alpha_{1}$ and $\alpha_{2}$ so as to meet a desired ratio of the expected responses of the two species contributions: $\left(\Delta G_{x_{1}}^{R},\Delta G_{x_{2}}^{R}\right)=c\left(k_{1},k_{2}\right)$.

When the expected responses to selection after recombination of selected candidates are not assessable (see preceding paragraphs), the index weights can alternatively be chosen in order to meet a desired ratio of expected responses of species contributions before recombination of selected candidates $\left(\delta_{x_{1}}^{R},\delta_{x_{2}}^{R}\right)=c\left(k_{1},k_{2}\right)$, provided that the same selection intensity is applied in both species. Index weights $\alpha_{1}^{\prime }$ and $\alpha_{2}^{\prime }$ are then the solutions of the following system of two equations with two unknown parameters:

$$\{\begin{array}{cc} \alpha_{1}^{\prime }\sigma_{G_{x_{1}}}^{2}+{\alpha^{\prime }}_{2}Cov\left(G_{x_{1}},G_{x_{2}}\right) & =k_{1}\\ {\alpha^{\prime }}_{1}Cov\left(G_{x_{1}},G_{x_{2}}\right)+{\alpha^{\prime }}_{2}\sigma_{G_{x_{2}}}^{2} & =k_{2}. \end{array}$$

If half-sib or topcross progeny families are used and $\theta_{1}\varphi_{1}=\theta_{2}\varphi_{2}=1$, the ratio of expected responses of species contributions after recombination of selected candidates depart more or less from the ratio $k_{1}/k_{2}$ according to the magnitude of the variance-covariances of interaction effects ${\left(va\right)}_{1r2s}$ and ${\left(va\right)}_{2s1r}$.

### Selection for general mixture ability (SGMA)

#### Selection scheme:

**Figure 2:**
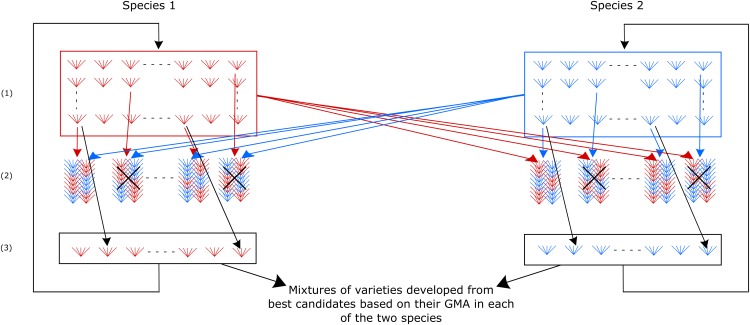
Parallel recurrent selections for General Mixture Ability (SGMA) in two species. (1): Populations of selection candidates at cycle *n*, (2): Experimental evaluation of mixtures of progeny families of selection candidates from one species with a bulk of all progeny families of candidates from the other species, (3): Recombination of the selected candidates.

As an alternative to the reciprocal scheme proposed by Wright (1985), we considered a selection scheme (Figure 2) aiming to improve two species for their General Mixture Ability in two parallel recurrent selection processes. At each selection cycle, progeny families of selection candidates from each species are tested in mixture with a balanced bulk of progeny families from all the candidates of the other species. With this selection scheme, the genetic component of the observed performance of the progeny family of a selection candidate is equal to its GMA. In each species, candidates selected at cycle *n* are recombined to generate the cycle n+1 population. At each selection cycle, progeny families from outstanding candidates in each species can be increased. Pairs of progeny families from these outstanding candidates can be mixed with a view to creating mixtures for farming usage. However, these mixtures should be experimentally tested since the testing system involved in the recurrent selection process does not enable to assess direct × associate interactions (or in other words the SMA effect).

#### Analytical model of performances in mixture:

Consider the selection in species 1 (*i.e.*, the left hand side part of Figure 2). $y_{1rm}$ is the performance of the $m^{th}$ observation of the mixture used to test the selection candidate *r* of species 1. Then, using the same notations as previously:

$$y_{1rm}=u_{1}+u_{2}+g_{1r}+\epsilon_{1rm},\;\text{with}\;\epsilon_{1rm}\to \left(0,\sigma_{\epsilon_{1}}^{2}\right).$$

$y_{1rm}$ can also be expressed as:

$$y_{1\mathrm{rm}}=x_{11\mathrm{rm}}+x_{21\mathrm{rm}},$$

where $x_{11\mathrm{rm}}=u_{1}+v_{1r}+e_{11\mathrm{rm}}$ is the observed contribution to the performance of the mixture of the progeny family from candidate *r* of species 1, $x_{21\mathrm{rm}}=u_{2}+a_{1r}+e_{21\mathrm{rm}}$ is the observed contribution to the performance of the mixture of the bulk of progeny families from all candidates of species 2 and $e_{11\mathrm{rm}}+e_{21\mathrm{rm}}=\epsilon_{1\mathrm{rm}}$.

#### Definition of a selection criterion:

A linear selection index can be set up to weight the contributions of the two species to the observed performance of tested mixtures. Considering selection in species 1, the selection index for the candidate 1r can be set as $I_{1r.}=\alpha_{11}x_{11r.}+\alpha_{21}x_{21r.}$.

The variance of this index is:

$$\begin{array}{l} \sigma_{I_{1r.}}^{2}=\alpha_{11}^{2}\sigma_{x_{11r.}}^{2}+\alpha_{21}^{2}\sigma_{x_{21r.}}^{2}+2\alpha_{11}\alpha_{21}Cov\left(x_{11r.},x_{21r.}\right)\\ =\alpha_{11}^{2}\sigma_{v_{1}}^{2}+\alpha_{21}^{2}\sigma_{a_{1}}^{2}+2\alpha_{11}\alpha_{21}Cov\left(v_{1},a_{1}\right)+\frac{1}{M_{1}}\left(\alpha_{11}^{2}\sigma_{e_{11}}^{2}+\alpha_{21}^{2}\sigma_{e_{21}}^{2}+2\alpha_{11}\alpha_{21}Cov\left(e_{11rm},e_{21rm}\right)\right) \end{array}$$

where $M_{1}$ is the number of observations of progeny families from selection candidates in species 1.

If $\alpha_{11}=\alpha_{21}=1$, then $I_{1r.}=y_{1r.}$. The selection criterion is then the observed performance of the mixture testing the progeny family of candidate 1r. Its variance can be written as:

$$\sigma_{y_{1r.}}^{2}=\sigma_{g_{1}}^{2}+\frac{1}{M_{1}}\sigma_{\epsilon_{1}}^{2}.$$

#### General expressions of expected responses to selection whatever type of progeny families is used:

Let $i_{1}$ be the selection intensity applied to selection in species 1. Under the assumption of negligible additive × additive epistasis, the expected response to selection of the two-species mixture performance from cycle *n* to cycle *n+1* is:

$$\Delta G_{1}^{G}=\theta_{1}\frac{i_{1}}{\sigma_{I_{1r.}}}Cov\left(I_{1r.},A_{g_{1r}}^{T_{1}}\right),$$

the expected response of the contribution of species 1 to the performance of the mixture is:

$$\Delta G_{x_{11}}^{G}=\theta_{1}\frac{i_{1}}{\sigma_{I_{1r.}}}\mathrm{Cov}\left(I_{1r..},A_{v_{1r.}}^{T_{1}}\right)$$

and that of species 2 is:

$$\Delta G_{x_{21}}^{G}=\theta_{1}\frac{i_{1}}{\sigma_{I_{1r.}}}Cov\left(I_{1r.},A_{a_{1r}}^{T_{1}}\right).$$

#### Expected responses to selection when half-sib or topcross progeny families are used:

For selection in species 1 and using the same notations as previously, the expected response to selection of the contribution of species 1 to the performance of the mixture is:

$$\Delta G_{x_{11}}^{G}=\theta_{1}\varphi_{1}\frac{i_{1}}{\sigma_{I_{1r.}}}\left(\alpha_{11}\sigma_{v_{1}}^{2}+\alpha_{21}Cov\left(a_{1},v_{1}\right)\right)$$

and that of species 2 is:

$$\Delta G_{x_{21}}^{G}=\theta_{1}\varphi_{1}\frac{i_{1}}{\sigma_{I_{1r.}}}\left(\alpha_{11}Cov\left(v_{1},a_{1}\right)+\alpha_{21}\sigma_{a_{1}}^{2}\right).$$

The variance-covariances of direct and associate effects (which are genetically additive) are straightforwardly assessable from the genetic variance-covariances of species contributions (and consequently from the test of progeny families in mixture implemented in the frame of the selection scheme). Thus:

$$\Delta G_{x_{11}}^{G}=\theta_{1}\varphi_{1}\frac{i_{1}}{\sigma_{I_{1r.}}}\left(\alpha_{11}\sigma_{G_{x_{11}}}^{2}+\alpha_{21}Cov\left(G_{x_{11}},G_{x_{21}}\right)\right)$$

and

$$\Delta G_{x_{21}}^{G}=\theta_{1}\varphi_{1}\frac{i_{1}}{\sigma_{I_{1r.}}}\left(\alpha_{11}Cov\left(G_{x_{11}},G_{x_{21}}\right)+\alpha_{21}\sigma_{G_{x_{21}}}^{2}\right)$$

where $G_{x_{11}}$ and $G_{x_{21}}$ are the genetic components of $x_{11r.}$ and $x_{21r.}$, respectively.

#### Expected responses to selection cumulated over the two selection processes:

Considering selection in species 2, mirroring expressions can be developed for the expected response to selection of the two-species mixture ($\Delta G_{2}^{G}$) and for the expected responses of contributions to the performance of the mixture of species 1 ($\Delta G_{x_{12}}^{G}$) and 2 ($\Delta G_{x_{22}}^{G}$). Assuming that selection is carried out at the same pace in the two species, expected responses from the two parallel selection processes can be summed.

$$\Delta G^{G}=\Delta G_{1}^{G}+\Delta G_{2}^{G},$$

$$\Delta G_{x_{1}}^{G}=\Delta G_{x_{11}}^{G}+\Delta G_{x_{12}}^{G}$$

and

$$\Delta G_{x_{2}}^{G}=\Delta G_{x_{21}}^{G}+\Delta G_{x_{22}}^{G}.$$

If the selection criterion is the observed performance of the tested mixtures in each of the two selection processes (*i.e.*, $y_{1r.}$ for selection in species 1 and $y_{2s.}$ for selection in species 2), then $\Delta G_{1}^{G}$, $\Delta G_{x_{11}}^{G}$ and $\Delta G_{x_{21}}^{G}$ (alternatively $\Delta G_{2}^{G}$, $\Delta G_{x_{22}}^{G}$ and $\Delta G_{x_{12}}^{G}$) are the expected responses of GMA, direct effect and associate effect in species 1 (alternatively species 2), respectively.

#### Tuning the selection index to meet a targeted ratio of expected responses of species contributions:

The two selection indices can be tuned in order to meet a certain ratio of expected responses of species contributions to the performance of the mixture cumulated over the two parallel selection processes $\left(\Delta G_{x_{1}}^{G},\Delta G_{x_{2}}^{G}\right)=c\left(k_{1},k_{2}\right)$. Assuming that additive variance-covariances of mixture model effects can be assessed, there is no unique set of the four index weights meeting the targeted objective. A sensible option is then to use the set providing the highest cumulated expected response to selection of the mixture performance $\Delta G^{G}$. The problem of finding this particular set of weights can be solved by implementing an algorithm of nonlinear constrained optimization.

### Correlative responses of performances in mixture to selection on pure stand performances

#### Selection scheme:

**Figure 3:**
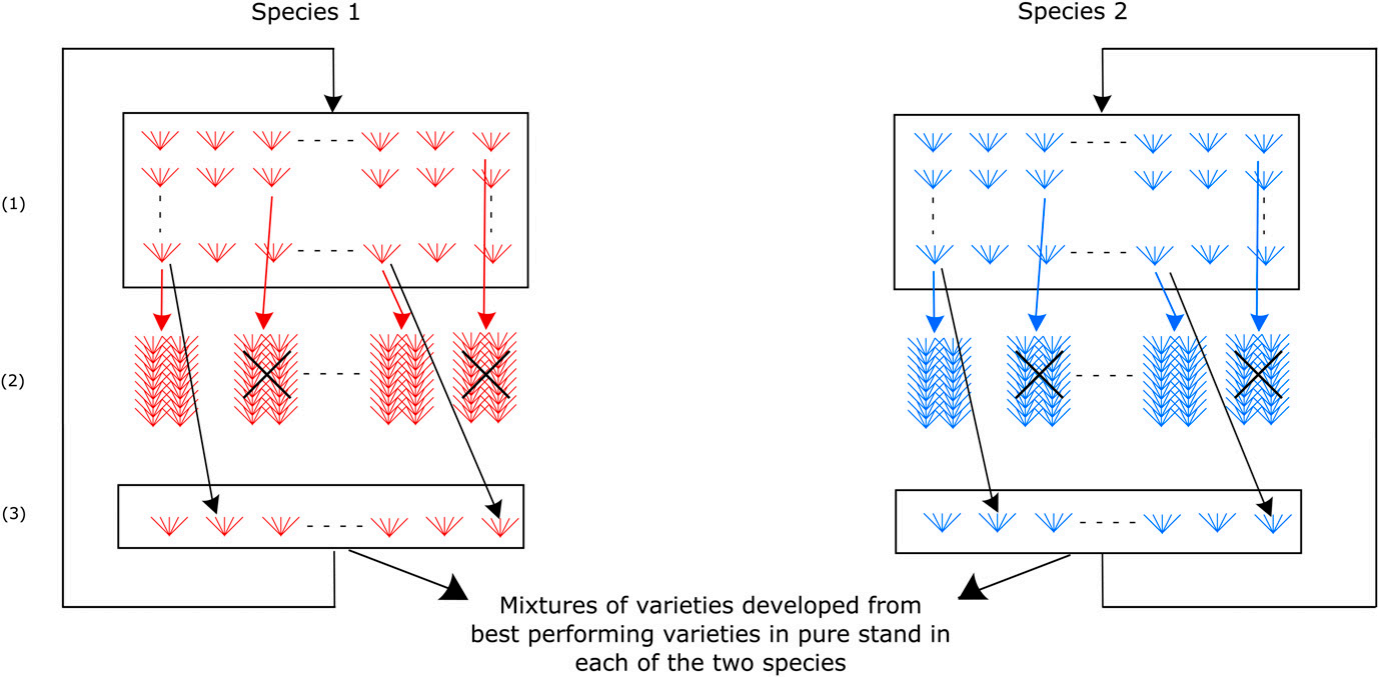
Parallel recurrent selections for pure stand performance in two species. (1): Populations of selection candidates at cycle *n*, (2): Experimental evaluation in pure stands of the progeny families of selection candidates, (3): Recombination of the selected candidates.

Recurrent selection is commonly used by plant breeders to improve the agro-economic value of plant species grown in monoculture. In this case, selection is usually based on the performance in pure stands of progeny families of selection candidates (Figure 3). The best candidates in pure stand conditions are straightforwardly used to develop cultivars adapted to monoculture. It is of course possible to grow cultivars selected in pure stands in a species mixture although they were not selected for such usage. More interestingly, the selection criterion used to select in pure stands could be an index of the agro-economic value in pure stands and of other traits recorded in pure stands and contributing to desirable performances in species mixture. Selection in pure stands could even focus only on traits contributing to performances in mixture, thus becoming an indirect selection for performances in mixture.

#### Analytical model:

Consider selection in pure stands in species 1. $y_{p_{1rm}}$ is the $m^{th}$ observation in pure stands of the progeny family of candidate to selection *r* of species 1 at cycle *n*. $y_{p_{1rm}}=u_{1}{}^{\prime }+p_{1r}+\epsilon_{p_{1rm}}$, where $u_{1}{}^{\prime }$ is the average effect of species 1 in pure stands and $p_{1r}$ is the genetic effect in pure stands of the progeny family of candidate *r* of species 1 with $p_{1r}\to \left(0,\sigma_{p_{1}}^{2}\right)$ and $\epsilon_{p_{1rm}}\to \left(0,\sigma_{\epsilon_{p_{1}}}^{2}\right)$. Assuming $M_{1}{}^{\prime }$ observations of the progeny family from each candidate to selection, the variance of the selection criterion $y_{p_{1r.}}$ is:

$$\sigma_{y_{p_{1r.}}}^{2}=\sigma_{p_{1}}^{2}+\frac{1}{M_{1}{}^{\prime }}\sigma_{\epsilon_{p1}}^{2}.$$

#### General expressions of expected responses to selection:

Consider the correlative responses that selection in pure stands in species 1 would provide if progeny families of offsprings of candidates selected at cycle *n* in pure stands were tested at cycle n+1 in the mixture conditions of the experimental design of SRMA or SGMA. Whatever the type of T1 progeny families of offsprings tested at cycle n+1, the expected correlative response of the contribution of species 1 to the performance of the mixture would be:

$$\Delta G_{x_{11}}^{P}=\theta_{1}^{\prime }\frac{i_{p_{1}}}{\sigma_{y_{p_{1r.}}}}Cov\left(p_{1r},A_{v_{1r}}^{T1}\right),$$

that of species 2 would be:

$$\Delta G_{x_{21}}^{P}=\theta_{1}^{\prime }\frac{i_{p_{1}}}{\sigma_{y_{p_{1r.}}}}Cov\left(p_{1r},A_{a_{1r}}^{T1}\right)$$

and the expected correlative response of the performance of the mixture would be:

$$\Delta G_{1}^{P}=\Delta G_{x_{11}}^{P}+\Delta G_{x_{21}}^{P}$$

where $\theta_{1}^{\prime }$ equals 1 or 2 according to whether selection in pure stands in species 1 is applied to one or two sexes,

and $A_{v_{1r}}^{T1}$ and $A_{a_{1r}}^{T1}$ are the average additive values inherited by the offsprings of candidate 1r at cycle *n+1*.

#### Expected correlative responses to selection of mixtures of half-sib or topcross progeny families:

If the T1 progeny families tested at cycle n+1 were half-sib or topcross progeny families, the expected correlative responses of species contributions to the performance of the mixture would be:

$$\Delta G_{x_{11}}^{P}=\theta_{1}{}^{\prime }\varphi_{1}\frac{i_{p_{1}}}{\sigma_{y_{p_{1r.}}}}Cov\left(p_{1r},v_{1r}\right)$$

and

$$\Delta G_{x_{21}}^{P}=\theta_{1}{}^{\prime }\varphi_{1}\frac{i_{p_{1}}}{\sigma_{y_{p_{1r.}}}}Cov\left(p_{1r},a_{1r}\right)$$

where $v_{1r}$ and $a_{1r}$ are the direct and associate effects, respectively, that would have a T1 progeny family of candidate r from species 1 in mixture conditions and $\varphi_{1}$ refers to the kind of T1 progeny families that would be used in mixture. Note that these expressions of expected responses to selection remain valid whatever the kind of progeny families used for testing in pure stands.

#### Expected responses to selection cumulated over the two parallel selection processes in pure stands:

With selection in pure stands in species 2, mirroring expressions can be developed for the expected correlative responses of the contributions to the performance of the mixture of species 1 ($\Delta G_{x_{12}}^{P}$) and 2 ($\Delta G_{x_{22}}^{P}$) and of the performance of the mixture ($\Delta G_{2}^{P}$). Assuming that selection in pure stands is carried out in parallel and at the same pace in the two species, the correlative responses of performances in mixture expected from the two selection processes can be summed:

$$\Delta G^{P}=\Delta G_{1}^{P}+\Delta G_{2}^{P},$$

$$\Delta G_{x_{1}}^{P}=\Delta G_{x_{11}}^{P}+\Delta G_{x_{12}}^{P}$$

and

$$\Delta G_{x_{2}}^{P}=\Delta G_{x_{21}}^{P}+\Delta G_{x_{22}}^{P}.$$

### Numerical comparisons of selection methods for expected responses of performances in mixture

#### Basic settings:

We carried out numerical comparisons of selection methods in the case where the progeny families used for testing in mixture are half-sib or topcross progeny families. We used the expressions of expected responses to selection set up for these two types of progeny families in the preceding paragraphs on the assumptions of disomic inheritance and negligible epistasis effects. We fixed $\theta_{1}\varphi_{1}=\theta_{2}\varphi_{2}=1$; this would correspond to the case where the progeny families used for tests in mixtures with the SRMA and SGMA schemes are either half-sib progeny families and selected half-sib progeny families are intercrossed or are topcross progeny families and selected candidates or their S1 progeny families are intercrossed. Selection in pure stands was assumed to apply to the same number of sexes (*i.e.*, $\theta_{1}{}^{\prime }=\theta_{1}$ and $\theta_{2}{}^{\prime }=\theta_{2}$). We compared the selection methods for equal experimental resources (*i.e.*, equal number of field plots for testing the progeny families of selection candidates from the two species whatever the selection method). We assumed that SRMA was implemented in order to maximize the selection intensity and thus that the progeny families of selection candidates from each species were tested in mixture with the progeny family of only one candidate from the other species. Assuming an equal number of observations (replicates) of tested progeny families with all selection methods, SRMA made it possible to test twice as many candidates in each species as the other selection methods. We considered that the number of candidates to be selected in each species was the same whatever the selection method. Practically, we fixed the selection rate to 10% with SRMA and to 20% with SGMA and selection in pure stands. Assuming a Gaussian distribution of the selection criterion, these selection rates corresponded to selection intensities 1.75 and 1.4, respectively. The number of observations (replicates) of tested progeny families was set to 3 for all selection methods. For a total of 900 available plots, these settings would correspond to test 300 selection candidates in each species for SRMA and 150 for SGMA and selection in pure stands, while selecting 30 candidates in each species whatever the selection method.

In the following numerical investigations, we set the variances of direct effects in the two species as $\sigma_{v_{1}}^{2}=\sigma_{v_{2}}^{2}=1$. We assumed that the observation (plot) error variance $\sigma_{\epsilon }^{2}$ was the same for the SRMA and the SGMA experimental designs and we set $\sigma_{\epsilon }^{2}=2\sigma_{v_{1}}^{2}$ (this did not assume a relationship between these two variances, but just intended to propose a likely order of magnitude of $\sigma_{\epsilon }^{2}$ against $\sigma_{v_{1}}^{2}$). The correlations between direct and associate effects, $\rho_{1}=\rho \left(v_{1},a_{1}\right)$ in species 1 and $\rho_{2}=\rho \left(v_{2},a_{2}\right)$ in species 2, were set equal ($\rho_{1}=\rho_{2}$) and ranging between −1 and +1. The correlation between the direct × associate interaction effects $\rho_{12}=\rho \left({\left(va\right)}_{12},{\left(va\right)}_{21}\right)$ was set equal to $\rho_{1}$ and $\rho_{2}$. For convenience purposes, the single value of $\rho_{1}=\rho_{2}=\rho_{12}$ is thereafter referred to as the ‘$\rho_{va}$ correlation’.

#### Comparison of the efficiency of SRMA and SGMA to improve the overall performance of the mixture:

We compared the responses to selection of the mixture performance expected with SRMA and SGMA when the selection criterion was the observed performance of tested mixtures. In the case of SRMA, pairs of progeny families were assumed to be selected on the basis of the performance of their mixture (*i.e.*, $y_{1r2s.}$ was the selection criterion). In the case of SGMA, progeny families were assumed to be selected in each species according to the performance of their mixture with a bulk of all progeny families from the other species (*i.e.*, $y_{1r.}$ and $y_{2s.}$ were the selection criterions for selection in species 1 and 2, respectively). Two values of the variance of associate effects were considered by setting $\sigma_{a_{1}}^{2}=\sigma_{a_{2}}^{2}=0.1$ or alternatively 0.5. The variance of the direct × associate interaction in the contribution of a species to the mixture performance was set as equal to half of the variance of the associate effect due to the other species, *i.e.*, $\sigma_{{\left(va\right)}_{12}}^{2}=\sigma_{a_{2}}^{2}/2$ and $\sigma_{{\left(va\right)}_{21}}^{2}=\sigma_{a_{1}}^{2}/2$. Within this range of variation of variance-covariances of mixture model effects, we computed the response to selection of the performance of the mixture expected with SRMA before and after recombination of selected candidates ($\delta G^{R}$ and $\Delta G^{R}$, respectively) and with SGMA ($\Delta G^{G}=\Delta G_{1}^{G}+\Delta G_{2}^{G}$).

#### Efficiency of selection in pure stands to improve the overall performance of the mixture:

We compared the correlative response of the mixture performance expected from the selection in pure stands in the two species to the direct response expected with the SGMA scheme in which the selection criterion was the observed performance of tested mixtures. With the selection in pure stands, progeny families were assumed to be selected according to the selection criterion $y_{p_{1r.}}$ in species 1 and $y_{p_{2s.}}$ in species 2. With SGMA, the selection criterion was assumed to be the observed performance of tested progeny families (*i.e.*, $y_{1r.}$ and $y_{2s.}$ for selection species 1 and 2, respectively). We set $\sigma_{a_{1}}^{2}=\sigma_{a_{2}}^{2}=0.1$ or alternatively 0.5. For selection in pure stands, we set the design heritabilities $\sigma_{p_{1}}^{2}/\sigma_{y_{p_{1r.}}}^{2}$ and $\sigma_{p_{2}}^{2}/\sigma_{y_{p_{2r.}}}^{2}$ equal to 0.6. The correlations between the genetic effects in pure stands and the direct effects in mixture $\nu_{1}=\rho \left(p_{1},\eta_{1}\right)$ and $\nu_{2}=\rho \left(p_{2},\eta_{2}\right)$ were set equal ($\nu_{1}=\nu_{2}$) and given three possible values -0.25, 0.5 or 0.75. The correlations between the genetic effects in pure stands and the associate effects in mixture $\omega_{1}=\rho \left(p_{1},a_{1}\right)$ and $\omega_{2}=\rho \left(p_{2},a_{2}\right)$ were set equal ($\omega_{1}=\omega_{2}$) and given three possible values -0.5, 0 or 0.5. Within this range of variation of variance-covariances of genetic effects, we computed the response to selection of the performance of the mixture expected with selection in pure stands ($\Delta G^{P}=\Delta G_{1}^{P}+\Delta G_{2}^{P}$) and SGMA ($\Delta G^{G}=\Delta G_{1}^{G}+\Delta G_{2}^{G}$).

#### Expected responses of species contributions by selecting on the overall performance of the mixture:

We considered three cases with different variances of associate effects in the two species: $\left(\sigma_{a_{1}}^{2},\sigma_{a_{2}}^{2}\right)=\left(0.5,0.1\right),\left(1,0.1\right)$ and (1,0.5). We additionally set different variances of interaction effects in the two species with $\sigma_{{\left(va\right)}_{12}}^{2}=\sigma_{a_{2}}^{2}/2$ and $\sigma_{{\left(va\right)}_{21}}^{2}=\sigma_{a_{1}}^{2}/2$. We assumed that the selection criterion was the observed performance of tested mixtures, *i.e.*, $y_{1r2s.}$ with SRMA and $y_{1r.}$ and $y_{2s.}$ with SGMA. Using these settings, we computed the expected responses to selection of the mixture performance and of species contributions in the case of SRMA ($\Delta G^{R}$, $\Delta G_{x_{1}}^{R}$ and $\Delta G_{x_{2}}^{R}$) and in that of SGMA ($\Delta G^{G}$, $\Delta G_{x_{1}}^{G}$ and $\Delta G_{x_{2}}^{G}$).

#### Control of expected responses of species contributions by index selection:

Using the same settings as in the preceding paragraph, we built indices aiming to achieve equal expected responses of the two species contributions with SRMA and SGMA. With SRMA, the index was set up in order to meet equal expected responses of species contributions before recombination of selected candidates ($\delta_{x_{1}}^{R}/\delta_{x_{2}}^{R}=1$), which is the only possibility when variance-covariances of mixture model effects are not assessable. With SGMA, we used the pair of indices $I_{1}$ and $I_{2}$ that provided the highest cumulated expected response to selection of the mixture performance ($\Delta G^{G}$) among those meeting the objective $\Delta G_{x_{1}}^{G}/\Delta G_{x_{2}}^{G}=1$. This best pair of indices was found using the function *fmincon* from the Matlab R2015a Optimization Toolbox (see Supplemental Material File S1, *Nonlinear constrained optimization*). With SRMA as well as with SGMA, the error ϵ of the analytical model of the observed performance of tested mixtures is the sum of two errors $e_{1}$ and $e_{2}$ pertaining to the two analytical sub-models of species contributions ($\epsilon =e_{1}+e_{2}$). For index selection implemented with each of the two selection methods, we considered that $\sigma_{e_{1}}^{2}=\sigma_{e_{2}}^{2}=\sigma_{\epsilon }^{2}$ and $Cov\left(e_{1},e_{2}\right)=-1/2\sigma_{\epsilon }^{2}$, which corresponds to a correlation between the two species components of the plot error $\rho \left(e_{1},e_{2}\right)$ equal to −0.5.

### Data availability

Supplemental materials are deposited via the GSA figshare portal. They comprise the following items:

Figures S1, S2 and S3
File S1 - Nonlinear constrained optimization.
Supplemental material available at figshare: https://doi.org/10.25387/g3.9938045.

## Results

### Comparison of the efficiency of SRMA and SGMA to improve the overall performance of the mixture

**Figure 4:**
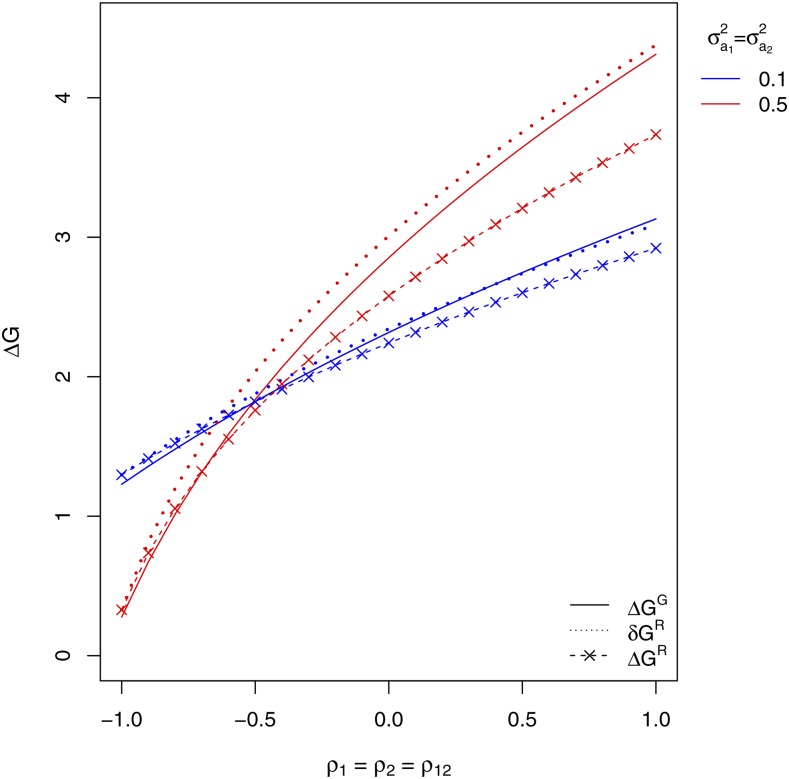
Comparison of the responses to selection of the mixture performance of two species expected from one cycle of recurrent selection for Reciprocal Mixture Ability (SRMA) and of parallel recurrent selections in the two species for General Mixture Ability with the other species (SGMA). With SRMA, the selection criterion was the observed performance of the mixture of pairs of progeny families (half-sib or topcross progeny families) of candidates from the two species. With SGMA, the selection criterion in each species was the observed performance of the mixture of the progeny family (half-sib or topcros progeny family) of a candidate with a bulk of all progeny families from the other species. The response to selection of the mixture performance expected with SRMA is displayed before recombination of selected candidates ($\delta G^{R}$) and after recombination ($\Delta G^{R}$). $\Delta G^{G}$ is the expected response to selection of the mixture performance cumulated over parallel selections in the two species with SGMA. The variance of direct effect was set equal to 1 in the two species. $\sigma_{a_{1}}^{2}$ and $\sigma_{a_{2}}^{2}$ are the variances of associate effects in species 1 and 2, respectively. $\rho_{1}$ and $\rho_{2}$ are the correlations between direct and associate effects in species 1 and 2, respectively. With SRMA, the variance of direct × associate interaction was set to 1/2 of the variance of associate effect in the variance of the species contributions to the observed performance of tested mixtures. $\rho_{12}$ is the correlation between the direct × associate interactions pertaining to each species contribution.

The response to selection of the mixture performance expected with SRMA and SGMA (Figure 4) increased with increasing values of the $\rho_{va}$ correlation between mixture model effects ($\rho_{1}=\rho_{2}=\rho_{12}$ in the case of SRMA, $\rho_{1}=\rho_{2}$ in the case of SGMA). With both selection methods, the variance of associate effects ($\sigma_{a_{1}}^{2}=\sigma_{a_{2}}^{2}$) had substantial impact on the expected response of the mixture performance. Increasing the value of the variance of associate effects increased the expected response of the mixture performance when the $\rho_{va}$ correlation was positive but decreased it when it was strongly negative ($\rho_{va}<-0.5$). Interestingly, the switch from negative to positive impact of increasing this variance occurred within the negative range of variation of the $\rho_{va}$ correlation, as it changed from highly to moderately negative values. The response of the mixture performance before recombination of selected candidates expected with SRMA was comparable to the cumulated response of the mixture performance expected with SGMA (Figure 4). However, when the variance of associate effects was set to the high value ($\sigma_{a_{1}}^{2}=\sigma_{a_{2}}^{2}=0.5$) and the $\rho_{va}$ correlation was greater than −0.5, the response after recombination of selected candidates expected with SRMA was clearly smaller than the response expected with SGMA (Figure 4).

### Efficiency of selection in pure stands to improve the overall performance of the mixture

**Figure 5:**
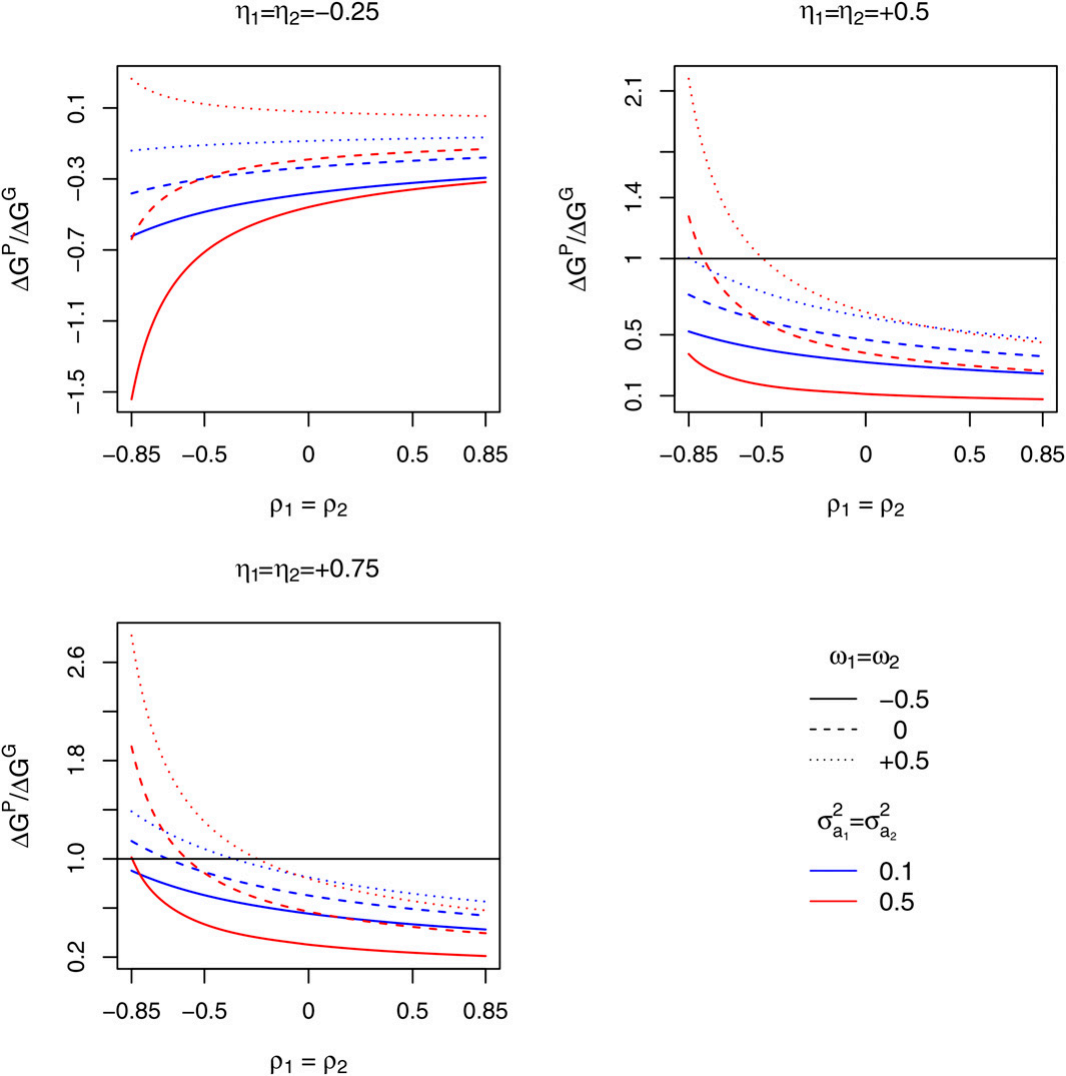
Comparison of the correlative response of the mixture performance of two species expected from one cycle of parallel recurrent selections in the two species for pure stand performance of progeny families ($\Delta G^{P}$) and of the direct response expected from one cycle of parallel recurrent selections in the two species for General Mixture Ability (SGMA) of progeny families with the other species ($\Delta G^{G}$). The ratio $\Delta G^{P}/\Delta G^{G}$ is plotted against the correlation between direct and associate effects set to the same value in the two species ($\rho_{1}=\rho_{2}$). The variance of direct effect was set equal to 1 in the two species. $\sigma_{a_{1}}^{2}$ and $\sigma_{a_{2}}^{2}$ are the variances of associate effects in species 1 and 2, respectively. $\eta_{1}$ and $\eta_{2}$ are the correlations between pure stand performance and direct effect in mixture for progeny families of candidates of species 1 and 2, respectively. $\omega_{1}$ and $\omega_{2}$ are the correlations between pure stand performance and associate effect in mixture for progeny families of candidates of species 1 and 2, respectively.

We fixed the variance of direct effects to be larger than that of associate effects. Therefore, the correlative response of the mixture performance expected with the selection in pure stands was negative when the pure stand performances were negatively correlated to the direct effects ($\eta_{1}=\eta_{2}=-0.25$), except when the pure stand performances were positively correlated to the associate effects ($\omega_{1}=\omega_{2}=0.5$) and the variance of associate effects was set to the high value ($\sigma_{a_{1}}^{2}=\sigma_{a_{2}}^{2}=0.5$) (Figure 5). The correlative response of the mixture performance expected with selection in pure stands was equal to, or larger than, the direct response expected with SGMA only when the correlations between direct and associate effects ($\rho_{1}=\rho_{2}$) were quite negative and the pure stand performances were positively correlated to the direct effects in mixture ($\eta_{1}=\eta_{2}=0.5$ or 0.75). It was also necessary that the pure stand performances were positively correlated to the associate effects in mixture and, if $\eta_{1}=\eta_{2}=0.5$, that the variances of associate effects were set to the high value (Figure 5). Note that similar trends could have been found by comparing the correlative response to selection in pure stands to the direct response obtained with SRMA, since only a relatively small difference was noticed between SRMA and SGMA for the response to selection of the mixture performance (see preceding paragraph).

### Expected responses of species contributions by selecting on the overall performance of the mixture

**Figure 6:**
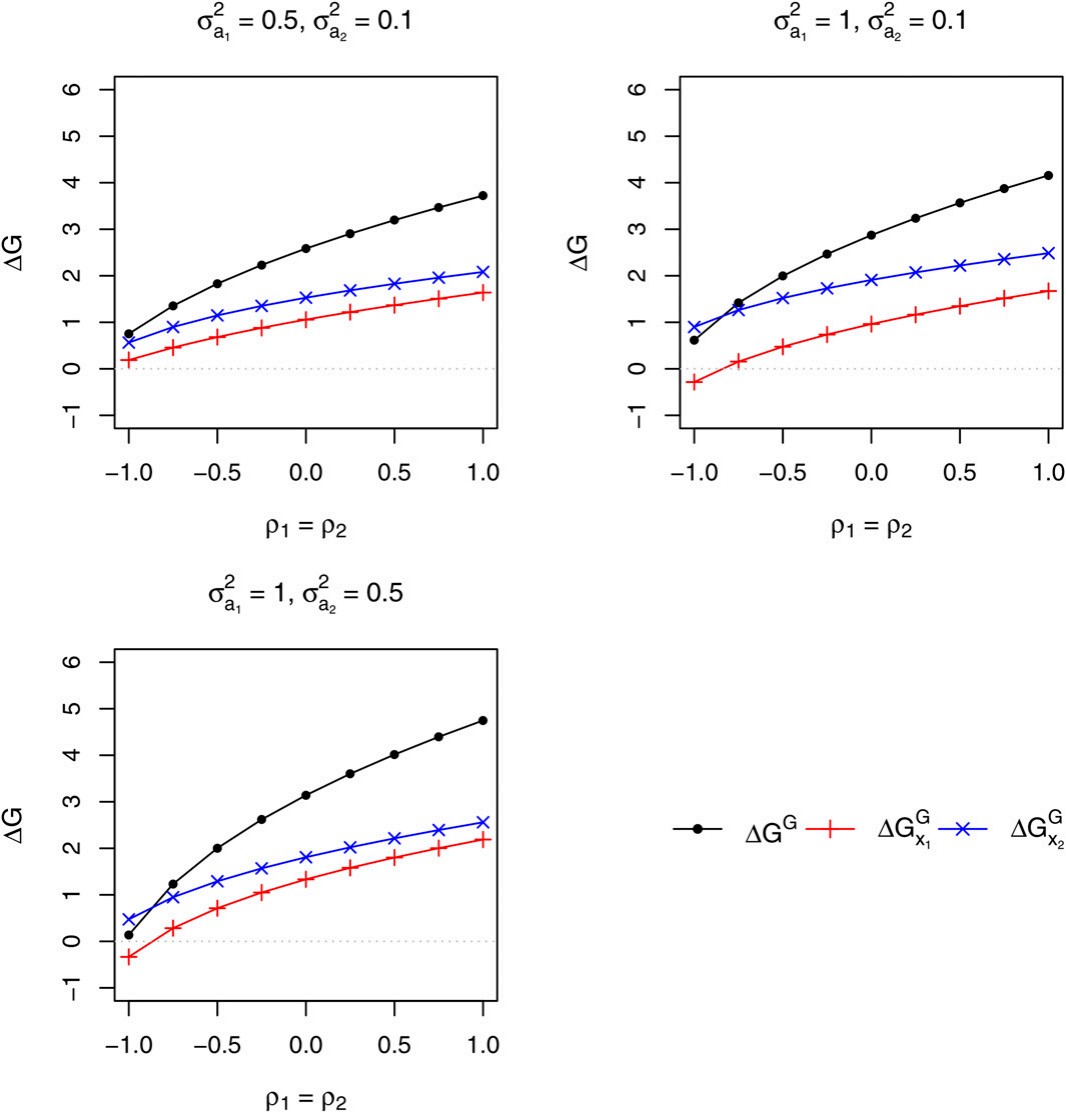
Responses to selection of the mixture performance of two species ($\Delta G^{G}$) and of species contributions to the mixture performance ($\Delta G_{x_{1}}^{G}$ and $\Delta G_{x_{2}}^{G}$) expected after one cycle of parallel recurrent selections in the two species for General Mixture Ability with the other species (SGMA). In each species, the selection criterion was the observed performance of the mixture of the progeny family (half-sib or topcros progeny family) of a selection candidate with a bulk of all progeny families from the other species. The variance of direct effect was set equal to 1 in the two species. $\sigma_{a_{1}}^{2}$ and $\sigma_{a_{2}}^{2}$ are the variances of associate effects in species 1 and 2, respectively. $\rho_{1}$ and $\rho_{2}$ are the correlations between direct and associate effects in species 1 and 2, respectively.

When the variances of mixture model effects were set differently in the two species, selecting only on the performance of the mixture with SRMA or SGMA naturally led to unequal expected responses of the contributions of the two species to the performance of their mixture (Figure S1 for SRMA and Figure 6 for SGMA). The choice we made of contrasting the two species ($\sigma_{a_{1}}^{2}>\sigma_{a_{2}}^{2}$) resulted in a genetic variance of species contribution that was smaller for species 1 than for species 2; the expected response of species 1 contribution was consequently smaller than that of species 2. Meanwhile, the variance of selection criteria ($\sigma_{y_{1r2s.}}^{2}$ for SRMA, $\sigma_{y_{1r.}}^{2}$ and $\sigma_{y_{2s.}}^{2}$ for SGMA) decreased with decreasing values of the $\rho_{va}$ correlation. Consequently, the difference between the expected responses of the two species contributions increased as the $\rho_{va}$ correlation decreased. This difference was of the same order of magnitude for SRMA and SGMA.

### Control of expected responses of species contributions using selection indices

#### Control of responses of species contributions with SRMA:

**Figure 7:**
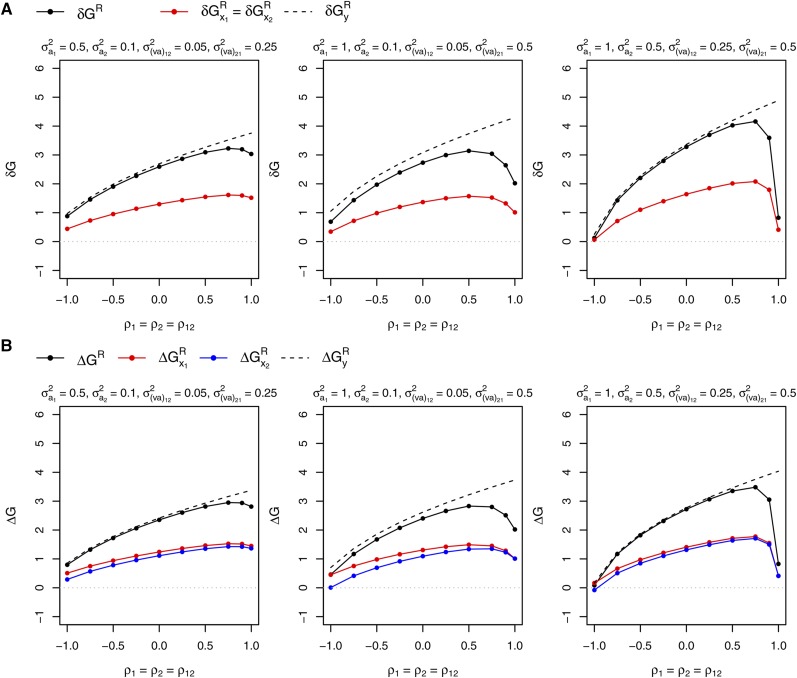
Responses to selection expected from one cycle of recurrent selection for Reciprocal Mixture Ability (SRMA) between two species aiming to equate the expected responses of the contributions of the two species to the performance of their mixture. The selection criterion was a linear combination (index) of the contributions of progeny families (half-sib or topcross progeny families) of pairs of candidates from the two species to the observed performance of their mixture. Index weight was tuned in order to equate the expected responses of species contributions before recombination of selected candidates ($\delta G_{x_{1}}^{R}/\delta G_{x_{2}}^{R}=1$). (A) Expected responses to selection of the performance of the mixture of the two species ($\delta G^{R}$) and of species contributions ($\delta G_{x_{1}}^{R}$ and $\delta G_{x_{2}}^{R}$) before recombination of selected candidates. (B) Expected responses to selection of the performance of the mixture of the two species ($\Delta G^{R}$) and of species contributions ($\Delta G_{x_{1}}^{R}$ and $\Delta G_{x_{2}}^{R}$) after recombination of selected candidates. The response to selection of the performance of the mixture expected when the selection criterion is the observed performance of tested mixtures is also reported before recombination of selected candidates ($\delta G_{y}^{R}$ on graphs (A)) and after recombination ($\Delta G_{y}^{R}$ on graphs (B)). The variance of direct effect was set equal to 1 in the two species. $\sigma_{a_{1}}^{2}$ and $\sigma_{a_{2}}^{2}$ are the variances of associate effects in species 1 and 2, respectively. $\sigma_{{\left(va\right)}_{12}}^{2}$ and $\sigma_{{\left(va\right)}_{21}}^{2}$ are the variances of direct × associate interactions in the variances of the contributions of species 1 and 2, respectively. The correlation between the two species components of the plot error was set to −0.5. See Figure 4 for the meaning of $\rho_{1}$, $\rho_{2}$ and $\rho_{12}$.

Selection on an index enabling to equate expected responses of species contributions before recombination of selected candidates (Figure 7A) resulted in expected responses of species contributions that differed only slightly after recombination of selected candidates (Figure 7B). The departure from equal responses was the largest, although still small, with negative values of the $\rho_{va}$ correlation. This departure was the most substantial in the case where the variances of direct × associate interactions were the most different between the two species contributions ($\sigma_{{\left(va\right)}_{12}}^{2}=0.05$ and $\sigma_{{\left(va\right)}_{21}}^{2}=0.5$). Since the variance of direct × associate interaction was smaller for species 1 contribution, the observed contribution of species 1 was overweighted in the index. Using unequal index weights led to a smaller expected response of the mixture performance than when selecting on the performance of the mixture only if the $\rho_{va}$ correlation was positive. With the values of variance-covariance of mixture model effects we set, the loss in expected response of the mixture performance was nevertheless small (except when the $\rho_{va}$ correlation was close to 1) ($\Delta G_{y}^{R}-\Delta G^{R}$ on Figure 7B). This loss was the largest when $\left(\sigma_{a_{1}}^{2},\sigma_{a_{2}}^{2},\sigma_{{\left(va\right)}_{12}}^{2},\sigma_{{\left(va\right)}_{21}}^{2}\right)=\left(1,0.1,0.05,0.5\right)$, *i.e.*, in the case where the variances of associate and interaction effects were the most different between the two species contributions. Implementing index selection appeared especially worthwhile when the $\rho_{va}$ correlation was negative. In this case, the loss in expected response of the mixture performance was negligible, even though the difference between responses of species contributions expected when selecting on the performance of the mixture was the largest with negative values of the $\rho_{va}$ correlation.

#### Control of responses of species contributions with SGMA:

**Figure 8:**
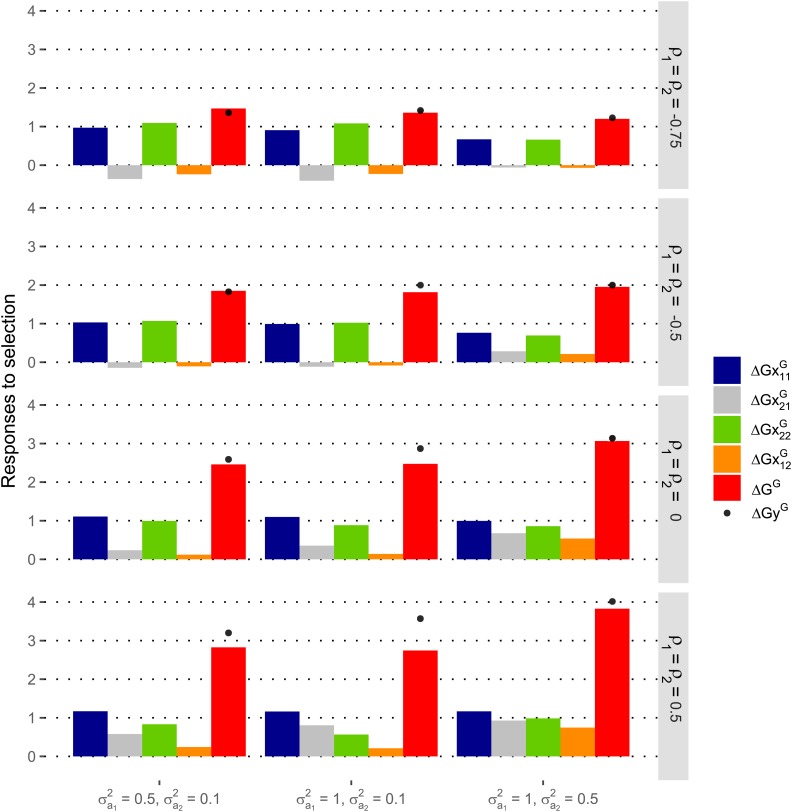
Responses to selection expected after one cycle of parallel recurrent selections in two species for General Mixture Ability with the other species (SGMA) aiming to equate the expected responses of the contributions of the two species to the performance of their mixture. In each species, the selection criterion of a candidate was a linear combination (index) of the observed contributions of its progeny family and of the bulk of all progeny families from the other species with which it is mixed. $\Delta G_{x_{11}}^{G}$ and $\Delta G_{x_{21}}^{G}$ are the expected responses to selection in species 1 of the contributions to the performance of the mixture of species 1 and 2, respectively. $\Delta G_{x_{22}}^{G}$ and $\Delta G_{x_{12}}^{G}$ are the expected responses to selection in species 2 of the contributions of species 2 and 1, respectively. The pair of indices applied to selections in species 1 and 2 was chosen as the one providing the highest cumulated expected response to selection of the performance of the mixture ($\Delta G^{G}$) among those providing equal cumulated expected responses of the contributions of the two species $\left(\Delta G_{x_{11}}^{G}+\Delta G_{x_{12}}^{G}\right)=\left(\Delta G_{x_{22}}^{G}+\Delta G_{x_{21}}^{G}\right)$. $\Delta G_{y}^{G}$ is the cumulated expected response to selection of the performance of the mixture when the selection criterion is the observed performance of tested mixtures in each of the two parallel selection processes. The variance of direct effect was set equal to 1 in the two species. $\sigma_{a_{1}}^{2}$ and $\sigma_{a_{2}}^{2}$ are the variances of associate effects in species 1 and 2, respectively. The correlation between the two species components of the plot error was set to −0.5. See Figure 4 for the meaning of $\rho_{1}$, $\rho_{2}$ and $\rho_{12}$.

The selection indices set up in the two species enabled to meet the target of cumulated expected responses over the two parallel selection processes (highest $\Delta G^{G}$ for $\Delta G_{x_{1}}^{G}/\Delta G_{x_{2}}^{G}=1$) without or with only a small loss on the expected response of the mixture performance ($\Delta G_{y}^{G}-\Delta G^{G}$ on Figure 8). This loss was substantial, although relatively small, only when the $\rho_{va}$ correlation was positive or null and $\left(\sigma_{a_{1}}^{2},\sigma_{a_{2}}^{2}\right)=\left(1,0.1\right)$. When the $\rho_{va}$ correlation equalled −0.75 and ($\sigma_{a_{1}}^{2}$, $\sigma_{a_{2}}^{2})=\left(0.5,0.1\right)$, the expected response of the mixture performance with index selection was even slightly higher than when selecting on the observed performance of tested mixtures. Setting equal weights to the observed contributions of the two species (*i.e.*, selecting on the observed performance of tested mixtures) may indeed not always maximize the expected response of the performance of the mixture depending on the relative importance of variance-covariances of error terms in the variances of indices. Since the variances of direct effects were set larger than the variances of associate effects in both species, they contributed more to the expected response to selection of the mixture performance ($\Delta G^{G}$). Consequently, the response of the contribution of the species under selection (*i.e.*, the response to selection of the direct effect in this species) was larger than the response of the contribution of the companion species (*i.e.*, the response to selection of the associate effect in the species under selection) with both selection processes (Figure 8). When the $\rho_{va}$ correlation was set negative, the best set of indices resulted in a slightly negative response of the contribution of the companion species with both selection processes, except when $\rho_{1}=\rho_{2}=-0.5$ and $\sigma_{a_{1}}^{2}=1$ and $\sigma_{a_{2}}^{2}=0.5$.

## Discussion

### Range of variation and genetics of mixture model effects

The number of selection candidates, the selection rates and the number of experimental replicates of candidate progeny families we set are in the order of magnitude of numbers commonly used in recurrent selection programs applied to broad-base populations in plant breeding. Regarding the experimental design for the assessment of progeny family performances in pure stands, the value we set for the design heritabilities ($\sigma_{p_{1}}^{2}/\sigma_{y_{p_{1r.}}}^{2}=\sigma_{p_{2}}^{2}/\sigma_{y_{p_{2r.}}}^{2}=0.6$) was in the range of values usually encountered for a quantitative trait fairly susceptible to environmental variation. The values of variance-covariances of mixture model effects we set were indeed fairly arbitrary. However, to our knowledge, experimental data are still not available to provide sound assessments of these variance-covariances in the conditions of the SRMA and SGMA experimental designs we considered. Nevertheless, setting for example $\rho_{1}=\rho_{2}=\rho_{12}=-0.5$, $\sigma_{a_{1}}^{2}=\sigma_{a_{2}}^{2}=0.5$ and $\sigma_{{\left(va\right)}_{12}}^{2}=\sigma_{{\left(va\right)}_{21}}^{2}=0.25$, the design heritability of the mixture performance would be equal to $\left(\sigma_{g_{1}}^{2}+\sigma_{g_{2}}^{2}+\sigma_{d_{12}}^{2}\right)/\sigma_{y_{1r2s.}}^{2}=0.73$ with the experimental design of SRMA and to $\sigma_{g_{1}}^{2}/\sigma_{y_{1r.}}^{2}=0.54$ with that of SGMA. Such heritabilities are also within a likely range. We assumed disomic inheritance and negligible epistasis effects and restricted our numerical investigations to the case in which half-sib or topcross progeny families are used for testing in mixture (*i.e.*, cases in which the variance-covariances of mixture model effects are genetically additive). The expressions of expected responses to selection we developed for half-sib and topcross progeny families would remain true in the case of polysomic inheritance if non-additive genetic effects were negligible (Gallais 2003). These expressions could also be adapted to the test of other types of progeny families when non-additive genetic effects can be assumed to be negligible. For instance, in the case of full-sib progeny families, one would have $\sigma_{v_{1}}^{2}=1/2\sigma_{A_{v_{1}}}^{2}$ and $Cov\left(v_{1r},A_{v_{1r}}^{T_{1}}\right)=1/4\sigma_{A_{v_{1}}}^{2}$ and in the case of S1 progeny families (families of offsprings of the self fertilization of candidates), $\sigma_{v_{1}}^{2}=\sigma_{A_{v_{1}}}^{2}$ and $Cov\left(v_{1r},A_{v_{1r}}^{T_{1}}\right)=1/2\sigma_{A_{v_{1}}}^{2}$ (Hallauer and Miranda 1981; Gallais 1989). Whatever the ploidy level, the type of progeny families used for testing and the implemented experimental design, it is at least always possible to set up selection indices to control expected responses to selection before recombination of selected candidates.

### Limits and opportunities for selection in pure stands

Selection in pure stands is expected to provide a correlative response on the mixture performance equal to, or higher than, the direct response obtained with SRMA and SGMA only in some particular situations. This only happens when the correlations between direct and associate effects in mixture are quite negative, which is however the case where SRMA and SGMA are expected to provide the smallest response to selection of the mixture performance. The selection in pure stands is then as efficient as, or more efficient than, SRMA and SGMA if the pure stand performances are positively correlated to both the direct and the associate effects in mixture. This situation is however not very likely if the direct and associate effects in mixture are negatively correlated. Note that substantial response of the mixture performance can still be expected with selection in pure stands (say 75% of the direct response expected with SRMA or SGMA) if the pure stand performances are positively correlated to the direct effects and uncorrelated to the associated effects. For efficient selection in pure stands, breeders should combine pure stand traits in a linear selection index which would present such correlations with direct and associate effects in mixture. This would however imply that the improvement of the performances in mixture is an explicit target of selection in pure stands. It should however be noted that selection in pure stands is always expected to be poorly efficient, compared to SRMA and SGMA, when the correlations between direct and associate effects are only slightly negative or are positive.

### SGMA has the best assets for the recurrent improvement of the species mixture performance

Our investigations for comparing the efficiency of SRMA and SGMA to improve the performance of the mixture indicated that both selection methods are able to take advantage of the variance of associate effects even if the correlations between direct and associate effects are moderately negative. With the settings we implemented, SGMA provided a higher expected response of the mixture performance than SRMA did when the correlations between direct and associate effects were set higher than −0.5 and the variance of associate effects was set to the largest value. With SRMA, the variance of the selection criterion is inflated by the variances of direct × associate interaction effects that are not inherited at the next selection cycle and this is not always compensated by the higher selection intensity which is possible with SRMA. With this last selection scheme, testing the progeny family of each candidate of a species in several mixtures of pairs of progeny families would require a reduction in the number of tested candidates at a constant total number of experimental plots and number of replicates of tested mixtures. At a constant number of selected candidates, the selection intensity and thus the expected response to selection of the mixture performance would be lessened. This drawback could be alleviated by reducing the number of replicates of tested mixtures but this would be at the expense of the capacity to accurately identify outperforming mixtures for farming usage, which is the main asset of SRMA. SGMA has furthermore the asset of providing a straightforward assessment of variance-covariances of direct and associate effects. It has however to be noted that SGMA would require twice as much seed amount of progeny families as SRMA for making experimental mixtures; this could also be a practical issue to take into account for choosing between SRMA and SGMA.

### The need to control responses of species contributions

Our results showed that selecting only on the performance of the mixture with SRMA and SGMA might lead to fairly large differences in the correlative responses of species contributions to the performance of the mixture when the variances of mixture model effects were set differently in the two species. This was especially the case when the single $\rho_{va}$ correlation value we set for the correlations between direct and associate effects, and between direct × associate interaction effects in the case of SRMA, was negative. With strongly negative values of the $\rho_{va}$ correlation, the correlative response of the contribution of the less responsive species could even be negative. Thus, selecting to improve the sole performance of the mixture may ultimately lead to undesirable proportions of the two species in the mixture. Differences in the correlative responses of species contributions are due to the fact that the species whose contribution has the largest genetic variance mechanically contributes more to the improvement of the performance of the mixture. These results notably emphasize the importance of the value of the correlation between the direct and associate effects with regard to the efficiency of selection for performances in mixture. This correlation is expected to be negative when compensation effects driven by competition are prevailing between the two species. However, sound experimental assessements of its value are still missing for genetic materials under selection. On the basis of an experimental design of grass-legume associations involving a small number of cultivars, Zannone *et al.* (1986) found a negative correlation. On the other hand, Williams *et al.* (2001) reported results from an experimental design of perennial ryegrass-white clover associations involving a small number of cultivars that imply a negative correlation under cutting conditions but a positive one under grazing conditions.

### Index selection as the mean to control responses of species contributions

In the case of the Reciprocal Mixture Ability scheme (SRMA) as well as in that of the General Mixture Ability scheme (SGMA), our results showed that selection indices that differently weighted the observed contributions of the two species to the performance of the mixture enabled to control the expected responses of species contributions without or with only a limited loss on the expected response of the mixture performance. This loss was particularly small or null when the correlation between mixture model effects ($\rho_{va}$ correlation) was set negative. The SRMA design we studied, in which the progeny families from each species are tested in mixture with a single progeny family from the other species, only makes possible to control responses to selection of species contributions before recombination of selected candidates. However, in the context of our numerical investigations, assuming that the variance-covariances of direct and associate effects are genetically additive and the variances of direct × associate interactions are relatively small, the ratio of expected responses of species contributions after recombination of selected candidates departed only slightly from the targeted ratio of expected responses before recombination. In the case of SGMA, pairs of selection indices that differently weight the observed species contributions in the two selection processes always enable to control the cumulated expected responses of species contributions after recombination of selected candidates, provided that the variance-covariances of direct and associate effects are genetically additive. It can be noted that the desired ratio of species contributions cumulated over the two parallel selection processes was not obtained by targeting this same ratio in each of the two selection processes in our numerical investigations.

In the numerical investigations that are presented in Figures 7 and 8, we set the correlation between errors of the two sub-models of species contributions as $\rho \left(e_{1},e_{2}\right)=-0.5$. Setting alternatively $\rho \left(e_{1},e_{2}\right)=0.5$ was not found to change the results significantly (Figures S2 and S3). The conclusions we draw about the effectiveness of index based selection are thus quite robust against possible variations of the correlation between errors on species contributions.

Index selection aiming to control the responses of species contributions requires to record actual species contributions in the experimental plots of progeny family mixtures. In cases like forage species mixtures for which the economically important feature is the harvested mixture biomass, assessing species contributions can be seen as an additional cost that could be invested elsewhere in the selection process. However, different technical options could be considered to limit this additional cost from the simple visual assessment of species proportions to the use of Near Infra Red Spectroscopy Reflectance (Cougnon *et al.* 2014; Karayilanli *et al.* 2016) or airborne imagery technologies (Lu and He 2017).

### SGMA should be ’reciprocal’

It is worthwhile to note that selection in a population, when carried out in the frame of the SGMA scheme, is fairly specific of the population involved in the companion species. The control of the ratio of species contributions is actually entirely subject to the mean values of the contributions of both populations. One could consider testing the progeny families from a population in a mixture with a ’tester’ population from the companion species that would not change from one selection cycle to another. This option would have the benefit of including specific interactions between this tester and the population under selection into direct and associate effects of the mixture model. However, the prospect for improvement with this single population selection could be substantially limited if direct and associate effects were negatively correlated in the population under selection. Our numerical investigations on the control of cumulated responses of species contributions with the use of selection indices in SGMA showed that a negative response of the associate effect in one species can be efficiently counterbalanced by a positive response of the direct effect obtained by selection in the other species. It should thus be much more efficient to submit populations from both species to parallel recurrent selections, hence making SGMA ’reciprocal’ to some extent.

### Selection in more than two species to improve their performances in mixture

Mixtures of plant species of agricultural interest may include more than two species (Meilhac *et al.* 2019). The Reciprocal and General Mixture Ability selection schemes could be extended to improve the mixture of more than two species. At each selection cycle, SRMA in *n* populations from different species would involve the testing of experimental mixtures including *n* progeny families, each one from a different population (or species). SGMA in *n* populations from different species would involve *n* parallel recurrent selection processes; in each selection process, progeny families from the population of a given species would be tested in mixtures with a bulk of all progeny families from the n−1 other populations (or species). In the Appendix, we provide the general expression of the response to selection of the performance of the mixture of the *n* species expected with SRMA and SGMA. In the case of SRMA, the variance of the selection criterion includes a number of variance components that would rapidly increase as the number of species increases. This variance may thus become excessively large, especially if the variances of SMA effects are substantial. From 3 species or more in the mixture, SRMA would require extremely high selection intensities to be efficient and this would imply unrealistically high numbers of tested progeny families. In the case of SGMA, the variance of the selection criteria only depends on one GMA variance term and of a fraction of residual variance and is thus more likely to stay within acceptable range. The General Mixture Ability selection scheme is therefore the most practical selection scheme when more than two species are to be improved for their performances in mixture. However, even in the case of this selection scheme, it is worthwhile to note that the genetic variance between tested progeny families (variance of GMA) depends on a substantial number of covariances between direct and associate effects and between associate effects. If compensation effects between species are important, most of these covariances may be negative; the genetic variance between tested progeny families may thus be small and the prospect of improvement of performances in mixture limited. In the frame of the General Mixture Ability selection scheme, index selection could also be implemented in order to target desired ratios of expected responses of the *n* species contributions by using methods of nonlinear constrained optimization.

### Conclusion

Litrico and Violle (2015) outlined the ecological features that are essential to take into account in order to optimize the production and stability of plant mixtures. In this paper, we used the framework of the theory of selection to investigate how recurrent selection schemes can be best adapted to reach the same objectives. Breeders may consider using elite populations that are already substantially improved for agro-economic value in pure stands in order to start recurrent selection programs to improve performances in species mixture. It is however possible that useful genetic variability for performances in mixture may be lost because of a selection for another kind of usage and because of a drift occurring concurrently with selection. In order to ultimately reach the best performances in mixture, it could thus be preferable to implement long term selection programs for performances in mixture starting from broad based populations including original genetic resources. An indirect selection based on the test of progeny families in pure stands may be first considered. However, our results showed that such indirect selection is worthwhile to implement only when the correlations between direct and associate effects are negative. Pure stand traits should be combined in a selection index in order to control the expected responses to selection of direct and associate effects in mixture. The selection intensity should be quite high so as to ensure sufficient responses to selection. Populations possibly improved by such indirect selection in pure stands could then be submitted to parallel recurrent selection processes for General Mixture Ability (SGMA). SGMA could efficiently control responses to selection of direct and associate effects in populations and could bring these populations up to elite level. SRMA could be worthwhile to consider at a later stage in the case of the improvement of mixtures of two species. This would have the benefit of including the testing of a large number of pairs of progeny families that could be straightforwardly selected to derive binary mixtures for farming usage; the variances of direct × associate effects would thus contribute to selecting the best mixtures for farming usage although they would not contribute to the recurrent improvement of populations. According to Muir (2005), it could be envisioned to set up genomic prediction models capable of predicting direct and associate effects even when these effects are not assessable from the analysis of variance of the SRMA design. Implementing such genomic predictions with SRMA could make it possible to control the responses to selection of direct and associate effects as efficiently as with SGMA, while keeping the typical SRMA asset of straightforward use of the best pairs for farming usage. When more than two species are to be improved for their performances in mixture, the General Mixture Ability selection scheme could be efficient, provided that compensation effects between species are not too prevalent.

## APPENDIX

### Reciprocal Mixture Ability and General Mixture Ability selection schemes to improve the mixture of populations from n species

#### Selection for Reciprocal Mixture Ability (SRMA):

There are *n* populations, each one from a different species, to reciprocally improve for the performance of their mixture. At each selection cycle, mixtures of *n* progeny families, each one from a different species, are tested.

Let $y_{l_{1}-l_{k}-l_{n}m}$ be the $m^{th}$ observation of the mixture performance of the *n* progeny families of selection candidates $l_{1},\ldots ,l_{k},\ldots ,l_{n}$ from species 1,…,k,…n. Assuming only first order interactions between mixture model effects, we have:

$$y_{l_{1}-l_{k}-l_{n}m}=\sum_{k}u_{k}+\sum_{k}g_{l_{k}}+\sum_{k\prime \#k}d_{l_{k}l_{k\prime }}+\epsilon_{l_{1}-l_{k}-l_{n}m}^{R}$$

where $g_{l_{k}}$ is the GMA of candidate $l_{k}$ from species *k* in mixture with the populations from the n−1 other species and $d_{l_{k}l_{k\prime }}$ is the SMA between candidate $l_{k}$ from species *k* and candidate $l_{k\prime }$ from species k′.

The selection criterion is the observed performance of tested mixtures of progeny families averaged over replicates, *i.e.* $y_{l_{1}-l_{k}-l_{n}.}$. Its variance is:

$$\sigma_{y_{R}}^{2}=\sum_{k}\sigma_{g_{k}}^{2}+\sum_{k\prime \#k}\sigma_{d_{kk\prime }}^{2}+\frac{1}{M}\sigma_{\epsilon_{R}}^{2}$$

where *M* is the number of observations (replicates) of each mixture of *n* progeny families. The expected response to selection of the performance of the mixture is then:

$$\Delta G^{R}=\sum_{k}\theta_{k}\frac{i_{k}}{\sigma_{y_{R}}}Cov\left(y_{l_{1}-l_{k}-l_{n}.},A_{g_{l_{k}}}^{T_{k}}\right)$$

where $A_{g_{l_{k}}}^{T_{k}}$ is the average additive genetic value for GMA inherited by offsprings of candidate $l_{k}$ from species *k* at the next selection cycle.

Assuming that all $\theta_{k}$ are equal to the same value $\theta_{R}$ and all $i_{k}$ are equal to the same value $i_{R}$, we have:

$$\Delta G^{R}=\theta_{R}\frac{i_{R}}{\sigma_{y_{R}}}\sum_{k}Cov\left(g_{l_{k}},A_{g_{l_{k}}}^{T_{k}}\right).$$

#### Selection for General Mixture Ability (SGMA):

There are *n* populations from different species to improve in *n* parallel recurrent selection processes. Each selection process aims to improve the GMA of the population of one of the species in mixture with the populations from the n−1 other species. At each selection cycle, each progeny family from the species under selection is tested in mixture with a bulk of all progeny families from the n−1 other species.

Let $y_{l_{k}m}$ be the $m^{th}$ observation of the performance of the mixture of the progeny family of candidate to selection $l_{k}$ from species *k* with all progeny families from the n−1 other species.

$$y_{l_{k}m}=\sum_{k}u_{k}+g_{l_{k}}+\epsilon_{l_{k}m}^{G}$$

The selection criterion is the observed performance of tested progeny family mixtures averaged over replicates, *i.e.* $y_{l_{k}.}$. Its variance is:

$$\sigma_{y_{G_{k}}}^{2}=\sigma_{g_{k}}^{2}+\frac{1}{M}\sigma_{\epsilon_{G_{k}}}^{2}.$$

The expected response to selection of the performance of the mixture from selection in species *k* is:

$$\Delta G_{k}^{G}=\theta_{k}\frac{i_{k}}{\sigma_{y_{G_{k}}}}Cov\left(y_{l_{k}.},A_{g_{l_{k}}}^{T_{k}}\right).$$

The expected response to selection of the performance of the mixture cumulated over the *n* selection processes is then:

$$\Delta G^{G}=\sum_{k}\Delta G_{k}^{G}.$$

Assuming that all $\theta_{k}$ are equal to the same value $\theta_{G}$ and all $i_{k}$ are equal to the same value $i_{G}$, we have:

$$\Delta G^{G}=\theta_{G}i_{G}\sum_{k}\frac{1}{\sigma_{y_{G_{k}}}}Cov\left(g_{l_{k}},A_{g_{l_{k}}}^{T_{k}}\right).$$

#### Comparison of responses to selection of the mixture performance expected with SRMA and SGMA:

$\sigma_{y_{R}}^{2}$ is larger than $\sigma_{y_{G_{k}}}^{2}$. As the number *n* of species increases, the difference between these variances increases. Thus, in order for SRMA to provide an expected response to selection of the mixture performance as large as that of SGMA, SRMA requires a much greater selection intensity.

Let us illustrate this in the following simplified case:

- $\theta_{R}=\theta_{G}$,
- $Cov\left(g_{l_{k}},A_{g_{l_{k}}}^{T_{k}}\right)=Cov\left(g_{l_{k\prime }},A_{g_{l_{k\prime }}}^{T_{k\prime }}\right)$ whatever species *k* and k′,
- $\sigma_{d_{kk\prime }}^{2}=0$ whatever species *k* and k′, *i.e.*, assuming negligible variance-covariances of first order interactions between mixture model effects,
- $\frac{1}{M}\sigma_{\epsilon_{R}}^{2}=\frac{1}{M}\sigma_{\epsilon_{G_{k}}}^{2}=0$, *i.e.*, assuming negligible fraction of error variance in the variance of the selection criterion.

In this simplified case, we would have:

$$\frac{\Delta G^{G}}{\Delta G^{R}}=n^{1/2}\frac{i_{G}}{i_{R}}.$$

Assume that SGMA applies with selection intensity $i_{G}=1.4$. To obtain the same expected response to selection of the mixture performance as that of SGMA, SRMA should apply with a selection intensity $i_{R}=2.42$ if n=3 (*i.e* with a selection rate equal to 2%). Such a selection rate would be fairly inapplicable if the number of selected candidates was to stay within a range sufficient to avoid drift (usually considered as around 30 candidates). The superiority of SGMA over SRMA would be even greater if the variance-covariances of interactions between mixture model effects were not negligible.

The expression of the variance of GMA is the same for SRMA and SGMA. Let $g_{l_{k}}$ be the GMA of the progeny family $l_{k}$ from species *k*, then:

$$g_{l_{k}}=v_{l_{k}}+\sum_{k\prime \#k}a_{k\prime l_{k}}$$

where $v_{l_{k}}$ is the direct effect of the progeny family $l_{k}$ of species *k* on the contribution to the performance of the mixture of species *k*, and $a_{k\prime l_{k}}$ is the associate effect of the progeny family $l_{k}$ on the contribution to the performance of the mixture of species k′.

The variance of GMA of species *k* is then:

$$\sigma_{g_{k}}^{2}=\sigma_{v_{k}}^{2}+\sum_{k\prime \#k}\sigma_{a_{k\prime k}}^{2}+2\sum_{k\prime \#k}Cov\left(v_{k},a_{k\prime k}\right)+2\sum_{k\prime \prime \#k\prime \#k}Cov\left(a_{k\prime \prime k},a_{k\prime k}\right).$$

$\sigma_{g_{k}}^{2}$ thus depends on a number of covariances between direct and associate effects and between associate effects. As the number of species making up the mixture increases, the number of covariances between mixture model effects increases more rapidly than the number of variances of direct effect and associate effects. If compensation trends between species are prevalent and fairly marked, most of these covariances are likely to be negative and the different $\sigma_{g_{k}}^{2}$ may thus be quite small. With SRMA as well as with SGMA, the expected response to selection of the performance of the mixture depends on the magnitude of the different $Cov\left(g_{l_{k}},A_{g_{l_{k}}}^{T_{k}}\right)$, which are smaller than the respective $\sigma_{g_{k}}^{2}$; the prospect of improvement of the performance of the mixture may thus be limited, even with SGMA, in the case of prevalence of compensation trends between species.
